# Chilling Stress Triggers VvAgo1-Mediated miRNA-Like RNA Biogenesis in *Volvariella volvacea*

**DOI:** 10.3389/fmicb.2020.523593

**Published:** 2020-09-15

**Authors:** Ming Gong, Ying Wang, Jinsong Zhang, Yan Zhao, Jianing Wan, Junjun Shang, Ruiheng Yang, Yingying Wu, Yan Li, Qi Tan, Dapeng Bao

**Affiliations:** ^1^Key Laboratory of Edible Fungi Resources and Utilization (South), National Engineering Research Center of Edible Fungi, Ministry of Agriculture, Institute of Edible Fungi, Shanghai Academy of Agricultural Sciences, Shanghai, China; ^2^Department of Food Science and Technology, College of Light Industry and Food Engineering, Nanjing Forestry University, Nanjing, China

**Keywords:** *Volvariella volvacea*, autolysis, Argonaute, chilling stress, RNAi

## Abstract

In *Volvariella volvacea*, an important species of edible mushroom, cryogenic autolysis is a typical phenomenon that occurs during abnormal metabolism. Analysis of gene expression profiling and qPCR showed that chilling stress (CS) significantly and continuously upregulated only one type of Argonaute in *V. volvacea*, i.e., VvAgo1. Structural and evolutionary analysis revealed that VvAgo1 belongs to the Ago-like family, and its evolution has involved gene duplication, subsequent gene loss, and purifying selection. Analysis of its interaction network and expression suggested that CS triggers VvAgo1-mediated miRNA-like RNA (milRNA) biogenesis in *V. volvacea* V23 but not in VH3 (a composite mutant strain from V23 with improved CS resistance). Small RNA sequencing and qPCR analysis confirmed that CS triggered the increased milRNA expression in V23 and not in VH3. The predicted target genes of the increased milRNAs were enriched in several pathways, such as signal transduction and ubiquitination. Heatmap analysis showed that CS altered the expression profile of milRNAs with their target genes related to signal transduction and ubiquitination in V23. Combined analysis of transcriptome and proteome data confirmed that most of the target genes of the increased milRNAs were not translated into proteins. Our observations indicate that CS might trigger VvAgo1-mediated RNAi to facilitate the cryogenic autolysis of *V. volvacea*.

## Introduction

*Volvariella volvacea*, also known as the straw mushroom or Chinese mushroom, is among the most extensively cultivated mushrooms in tropical and sub-tropical regions, but it requires relatively high temperatures (28–35°C) for vegetative growth and fruiting (Chen et al., 2003; Bao et al., 2013; Gong et al., 2016). Straw and cotton waste are the substrates used for industrialized cultivation of *V. volvacea*, making it the main vehicle for the utilization of straw as a biomass energy source. Routine storage at low temperatures (4°C) causes *V. volvacea* mycelia to undergo autolysis, and the fruiting body becomes soft, liquid, and even rotten (Chang, 1978). The short shelf-life of this mushroom hampers its distribution over long distances and limits its cultivation and popularization. Cryogenic autolysis of *V. volvacea* also indirectly affects the use of straw as an energy resource and has significant economic consequences, given that the annual output of *V. volvacea* on the Chinese mainland accounted for more than 80% of the global production of this commodity in 2010 (Bao et al., 2013). The issue of cryogenic autolysis is both an interesting scientific problem and a practical economic matter (Gong et al., 2016).

Cryogenic autolysis of *V. volvacea* is a response to chilling stress that is different from the cold shock response of many important edible fungi, such as Perigord black truffle (Zampieri et al., 2011), *Pleurotus eryngii* (Fu et al., 2016), and *Lentinula edodes* (Chum et al., 2008). The unique cryogenic autolysis of *V. volvacea* makes it a suitable model species to study the chilling stress response. Genomic sequencing and gene annotation of edible fungi, including *V. volvacea*, have facilitated the study of chilling stress-responsive genes involved in cryogenic autolysis (Bao et al., 2013; Gong et al., 2015, 2016, 2020).

Our previous cold-induced gene expression profile of *V. volvacea* showed that one type of Argonaute in *V. volvacea*, i.e., VvAgo1, is significantly upregulated (Bao et al., 2013). Argonaute (Ago) proteins can mediate target silencing by translational repression and de-adenylation coupled with mRNA decay, which has an impact on diverse biological processes (Sasaki and Tomari, 2012). Many Agos that respond to chilling stress (CS) are found in various plant species, such rice (Kapoor et al., 2008), wheat (Meng et al., 2013), and cucumber (Gan et al., 2017). The identification of VvAgo1 in response to CS (Bao et al., 2013) suggests that fungal Agos are responsible for chilling stress-response similar to plant Agos.

The transcriptional machinery responsible for the synthesis of fungal miRNA-like RNAs (milRNAs) was first identified in *Neurospora crassa* (Yang et al., 2013). Various small RNA pathways involving Dicer and Ago genes central to the RNA interference (RNAi) machinery exist in the entomopathogenic fungus *Metarhizium robertsii* (Meng et al., 2017). The discovery of milRNAs has been reported mainly in pathogenic fungus, which represents their direct interactions with plants and animals (Bai et al., 2015; Wang et al., 2018; Zeng et al., 2018). Recent research has identified milRNAs in the development of several saprophytic Basidiomycetes species, such as *Antrodia cinnamomea* (Lin et al., 2015), *Coprinopsis cinerea* (Lau et al., 2018), and *Ganoderma lucidum* (Shao et al., 2020), and some predicted target genes of the milRNAs identified in these species are associated with environmental stimulus. As RNA silencing is a sequence-specific gene regulation system conserved in eukaryotes (Sasaki and Tomari, 2012), the chilling stress-responsive feature of VvAgo1 (Bao et al., 2013) indicates that it may be involved in the cryogenic autolysis of *V. volvacea* via milRNA.

We obtained the VH3 strain by composite mutagenesis of the V23 strain; this strain has improved cold resistance compared to V23, and its mycelium can tolerate 4°C for 9–10 days (Han et al., 2004). Comparative research between VH3 and V23 after CS treatment helps to understand the molecular mechanisms of cryogenic autolysis of *V. volvacea*; therefore, we used these strains in this study.

## Materials and Methods

### Sample Preparation

The *V. volvacea* V23 strain was obtained from the China General Microbiological Culture Collection Center (Beijing) (no. CGMCC5.289). The VH3 strain is available from the Culture Collection Center at the Institute of Shanghai Academy of Agricultural Sciences. The mycelia of the fungi were first incubated on potato dextrose agar plates at 32°C for 4 days. They were then punched out and inoculated in 100 mL of potato dextrose broth (PDB) (BD, Sparks Glencoe, MD, United States) in a 250 mL Erlenmeyer flask. The flasks were placed on a rotary shaker at 150 rpm at 32°C for 4 days. The mycelia were harvested from liquid cultures by filtration and then exposed to 4°C for 0, 2, 4, 6, and 8 h. After being washed with sterile distilled water and patted dry with filter paper, the treated mycelia were immediately frozen in liquid nitrogen and stored at −70°C before analysis.

### qPCR Assays

The treated mycelia were collected and lyophilized, and total RNA was extracted using TRIzol reagent (Invitrogen) according to the manufacturer’s instructions. Details of the qPCR assay are described in a previous study (Gong et al., 2016). The primers used for qPCR are listed in Supplementary Table S1. All qPCR assays were performed in three independent experiments, producing comparable results. GAPDH was used as a reference to normalize the expression of target genes according to previous studies (Qiao et al., 2009; Gong et al., 2016). The relative quantification of transcripts was calculated using the comparative CT method (2^–ΔΔ*CT*^) calibrated to GAPDH. qPCR of milRNA was performed as previously described (Li et al., 2015). The primers used for qPCR of milRNA are listed in Supplementary Table S13. One-way ANOVA followed by the Tukey test for multiple comparisons was conducted using SigmaStat Windows Version 3.5 to evaluate significant differences between mean values among groups (*P* < 0.05).

### Family Size Analysis of Saprophytic Basidiomycetes Agos

Because basidiomycetes and ascomycetes fungi have independent evolutionary trajectories, 11 representative basidiomycetes species from various niches were selected for comparative genomic analysis of saprophytic basidiomycete (sAgos) (Gong et al., 2013, 2015, 2016). These include *Agaricus bisporus*, *Coprinopsis cinerea*, *Cryptococcus neoformans*, *Ganoderma lucidum*, *Schizophyllum commune*, *Phanerochaete chrysosporium*, *Tremella mesenterica*, *Trichosporon chiarellii*, *Ustilago hordei*, *Ustilago maydis*, and *V. volvacea*. The tandem concatenated sequences, which consisted of single-copy orthologous sequences, were then used to build a phylogenomic tree. Details of the construction of the phylogenetic tree can be found in a previous study (Bao et al., 2013).

Then, the constructed phylogenomic tree was used to analyze the size of the sAgos family. The Ago proteins in *V. volvacea* were annotated using InterproScan program^1^. Homologous sequences of the Ago proteins in *V. volvacea* were extracted from 11 representative basidiomycetes genomes based on BLAST search results (BLASTp, cut-off *e*-value ≤ 1e-15). Multigene families were identified from all Ago proteins obtained from selected genomes using SCPS tools with default settings (BLASTp, cut-off *e*-value ≤ 10–50) (Paccanaro et al., 2006). The multigene families obtained in this way were then analyzed for evolutionary changes in the size of the sAgos families using the CAFE program (De Bie et al., 2006).

### Phylogenetic Analysis

Eight representative basidiomycetes species genomes were searched for sequences homologous to VvAgo1 using BLAST (BLASTp, cut-off *e*-value ≤ 10–50). Together with a representative set of 98 eukaryotic Argonaute protein (eAgos) used in the phylogenetic tree of Ago proteins (Swarts et al., 2014), a total of 131 eAgos were used for multiple alignments of conserved blocks of MID and PIWI domains according to previous methods (Swarts et al., 2014). Sequences were aligned using Muscle at default parameters (Edgar, 2004). Maximum likelihood trees were inferred using PhyML v3.0 (Guindon et al., 2010) with the LG model (Le and Gascuel, 2008). Clade support was calculated using SH-like approximate likelihood ratio tests (aLRT) (Anisimova et al., 2011). PhyML analyses were performed using NNI tree topology searches with estimated Gamma shape parameters.

### Detection of Natural Selection

The ratio of non-synonymous substitutions (those causing amino acid alterations) to synonymous substitutions (silent) (ω = dN/dS) provides a sensitive measure of natural selection at the protein level. ω values of 1, >1, and <1 indicate neutral evolution, positive selection, and purifying selection, respectively. A *Z*-test of selection was used to detect natural selection. The variance in the difference was computed using a modified Nei–Gojobori method (assuming transition/transversion bias = 2) (Zhang et al., 1998). The analysis involved 12 nucleotide sequences, and all ambiguous positions with less than 70% site coverage were eliminated. Evolutionary studies were conducted in MEGA7 (Kumar et al., 2016).

Further analysis of the selection pressure acting on codons was carried out using the Datamonkey webserver^2^ (Delport et al., 2010). The study was performed using 12 nucleotide sequences. dN/dS was estimated using three different approaches, including single likelihood ancestor counting (SLAC), fixed effects likelihood (FEL), and internal branches fixed-effects likelihood (IFEL). The best model for nucleotide substitutions for different datasets, as determined through the available tool in the Datamonkey server, was used in this analysis.

### Analysis of Molecular Modeling and Protein–Protein Interaction Networks

SWISS-MODEL was used for three-dimensional homology modeling of VvAgo1 using its default parameters (Biasini et al., 2014). The crystal structure of human Argonaute-2 (PDB code: 5t7b) served as the structural template for modeling. Detailed results of the alignment between VvAgo1 and Argonaute-2 are shown in Supplementary Table S2. TopMatch^3^ was used for pairwise 3D alignment.

The protein–protein interactions of VvAgo1 were analyzed using *Coprinopsis cinerea* as the reference organism in the STRING database (Szklarczyk et al., 2015), as it is a close herb-decay relative of *V. volvacea* (Bao et al., 2013). A high confidence score (0.7) was selected for the interaction analysis.

### High Throughput Sequencing of Small RNA

Three independently grown mycelia of *V. volvacea* V23 or VH3 after cold treatment for 0 and 4 h were collected and lyophilized, and total RNA was extracted using the TRIzol reagent (Invitrogen, Carlsbad, CA, United States) according to the manufacturer’s instructions. RNA degradation and contamination were monitored on 1% agarose gels. Total RNA quantity and purity were determined by the OD_260/280_ ratio and checked using a NanoPhotometer^®^ spectrophotometer (Implen USA, Westlake Village, CA, United States). RNA integrity was assessed using an RNA Nano 6000 Assay Kit with the Agilent Bioanalyzer 2100 system (Agilent Technologies, Santa Clara, CA, United States).

Small RNA libraries were constructed using the NEB Next Multiplex Small RNA Library Prep Set for Illumina (New England Biolabs, Ipswich, MA, United States) according to the manufacturer’s instructions. Briefly, 1 μg of total RNA per sample after cold treatment for 0 and 4 h was ligated to a 3′ adapter and a 5′ adapter using Ligation Enzyme Mix. The resulting samples were reverse-transcribed using Superscript II reverse transcriptase. Amplification was executed for the PCR products. All steps were performed according to the manufacturer’s protocols. The sequencing library was then sequenced on a HiSeq platform (Illumina) by Shanghai Personal Biotechnology Cp. Ltd. Small RNA libraries were analyzed for quality control, and the average total size of inserts was approximately 140–150 bp. After filtering the raw data to obtain clean data, we used sequences of 18–36 nt length and then removed repetitive sequences in each sample to leave unique reads. Then, the unique reads were mapped to the *V. volvacea* genome and compared with the Rfam database for screening the four known types of ncRNAs, including rRNA, tRNA, snRNA, and snoRNA (mismatch ≤ 1). The remaining unaligned sequences were compared with the plant mature miRNA sequences in miRBase version 22 for obtaining milRNAs (mismatch ≤ 1). Mireap was used to predict new miRNAs from the remaining unaligned sequences.

DEGseq software (Wang et al., 2010) was used to normalize the data and assess significant differences in milRNA expression, and |fold change| > 2 and *P*-value < 0.05 were used as a threshold to determine significant differentially expressed milRNA genes. The target genes of milRNA were predicted using psRobot (Wu et al., 2012). Gene-annotation enrichment analysis of these genes was carried out using DAVID (Huang da et al., 2009) and STRING (Szklarczyk et al., 2015). Pathway and process enrichment analysis was carried out using Metascape (Zhou et al., 2019).

## Results

### VvAgo1 in Response to Chilling Stress

**FIGURE 1:**
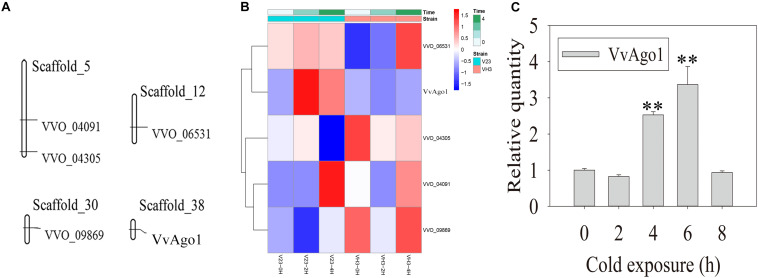
Genomic location of *V. volvacea* Agos and their gene expression under CS. **(A)** Genomic location of five Ago genes identified in the genomic scaffolds of *V. volvacea*. **(B)** Five Ago genes identified in *V. volvacea* and their expression at 2 and 4 h according to high-throughput sequencing analysis of mRNAs expressed in the mycelia of *V. volvacea* V23 strain and VH3 strain after cold exposure (4°C) for 0, 2, and 4 h (Bao et al., 2013; Gong et al., 2020). The details about the expressed values are provided in Supplementary Table S4. **(C)** qPCR analysis of VvAgo1 expression. Bars represent the mean ± standard deviation, and two asterisks indicate *P* < 0.001 relative to 0 h.

Five Ago genes were identified in the *V. volvacea* genome using InterProScan (Supplementary Table S3). Genomic distribution analysis showed that the five Ago genes occurred in four different scaffolds in *V. volvacea* (Figure 1A). One type of Ago (VVO_10604) in *V. volvacea* (VvAgo1) located in scaffold_38 was significantly and continuously expressed (| log2 (fold-change)| ≥ 1 and FDR < 0.001) in V23 instead of in VH3; the other four were not (Figure 1B and Supplementary Table S4). Further quantitative real-time PCR (qPCR) analysis confirmed that the expression of VvAgo1 was significantly and continuously upregulated after cold-treatment sessions of 4 and 6 h in V23 (Figure 1C; *P* < 0.001). The appearance of VvAgo1 peaked at 6 h, and it was 3.78-fold higher than the value at 0 h. Although VvAgo1 did not rise at 2 h, the trend toward its upregulation was apparent after cold treatment. qPCR confirmed that CS did not trigger the significantly upregulated expression of the other four Agos in V23 (Supplementary Figure S1). Our observations provided evidence that only VvAgo1 clearly responds to CS.

### Evolutionary Analysis of VvAgo1

**FIGURE 2:**
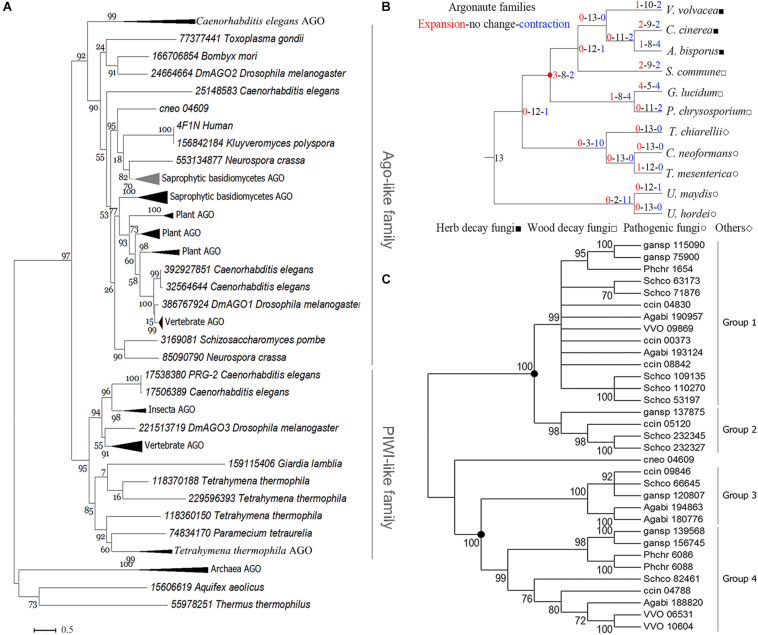
Phylogenetic analysis of the Ago family. **(A)** Phylogenetic analysis for the conserved blocks of MID and PIWI domains of a total 131 eukaryotic Ago proteins. Details about these compressed branches are provided in Supplementary Figure S2. **(B)** Analysis of changes in size and number of Ago family members in representative basidiomycetes. The red circle indicates the node for the divergence of sAgos. **(C)** Phylogenetic analysis of the Ago family in representative basidiomycetes. Only 70% of bootstrap values are shown. The solid circle indicates a gene duplication event. The topology of the protein phylogenetic tree was constructed via maximum-likelihood (ML) estimation (Bootstrap = 1000, LG model + Gamma5) using MEGA6 (Tamura et al., 2013). The following abbreviations are used: VVO, *Volvariella volvacea*; ccin, *Coprinopsis cinerea*; Agabi, *Agaricus bisporus*; Schco, *Schizophyllum commune*; phchr, *Phanerochaete chrysosporium*; gansp, *Ganoderma lucidum*, and cneo, *Cryptococcus neoforman*.

Phylogenetic analysis of eukaryotic Agos showed that the tree could be confidently divided into two major branches, the Ago-like family and the PIWI family (Figure 2A), in agreement with previous analyses (Swarts et al., 2014). The sAgos formed two lineages and belonged to the Ago-like family. The two sAgos lineages located in different groups indicated that ancient gene duplication led to their divergence.

The evolutionary analysis of the Ago family in 11 representative basidiomycete species showed that only mild changes occurred over their evolution (red circle in Figure 2B), because the net value was 1. The net amount at the nodes leading to the herb decay fungi (HDF) Agos and wood decay fungi (WDF) Agos was −2 and −3, respectively (Figure 2B). These data indicated that gene contraction occurred during the evolution of the sAgos family.

Further phylogenetic analysis revealed that the two independently evolving lineages in the sAgos, namely a common ancestor at the two nodes (see solid circle), diverged into four different sized groups (Figure 2C). Groups 1 and 2 consist of one sAgos lineage, while groups 3 and 4 consist of another sAgos lineage. Each group has at least three species, including HDF and WDF, which indicates that gene duplication occurred during sAgos evolution. Two recent gene duplication events marked by solid circles were detected in sAgo evolution (Figure 2C). The sizes of groups 2 and 3 were smaller than those of groups 1 and 4, indicating gene loss after gene duplication in these groups.

The independently evolving sAgo lineage including groups 3 and 4 was here named the VvAgo1 subfamily. Detection of natural selection of the VvAgo1 subfamily showed there to be purifying selection in the evolution of VvAgo1, which also occurred in the other HDF Agos. Selection pressure analysis showed several sites under positive selection in several WDF Agos (Supplementary Table S5). Further analysis indicated that an average of 422 (SEM = 56.42) negatively selected sites that were calculated using three different approaches of dN/dS ratio estimation were under purifying selection, compared with a few sites under positive selection detected by the three methods (Supplementary Table S6). Results indicated that purifying selection dominated the evolution of the VvAgo1 subfamily.

### Domain Analysis of VvAgo1

**FIGURE 3:**
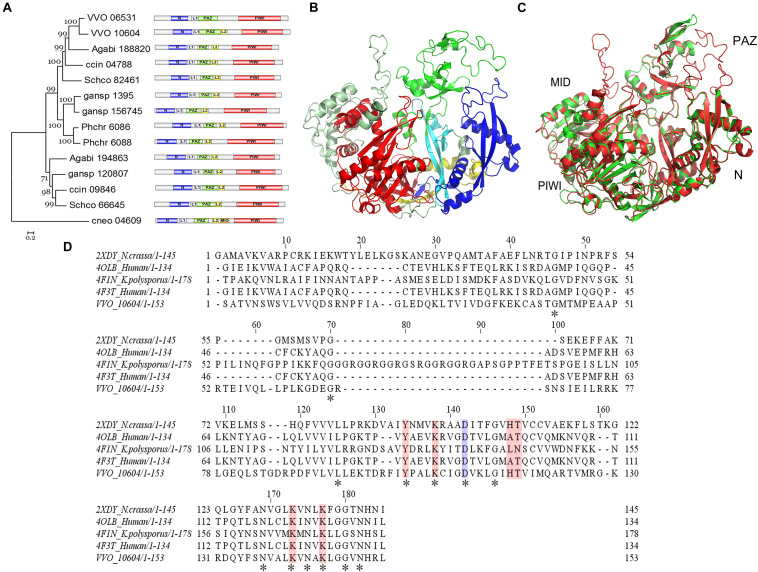
Functional domain and structure analysis of the VvAgo1 subfamily. **(A)** Analysis of the VvAgo1 phylogenetic tree. Only 70% of bootstrap values are shown. The solid circle indicates the node for the divergence of basidiomycetes Ago. VVO_10604 represents VvAgo1. Purple represents the N-terminal (N) domain; green represents the PIWI-Argonaute-Zwille (PAZ) domain; yellow represents a domain linker (L2); orange represents a middle (MID) domain; and red represents the P element-induced wimpy testis (PIWI) domain. **(B)** Structural analysis of VvAgo1. Different colors represent different domains according to **(A)**. **(C)** 3D view of superimposed structures of VvAgo1 and human Argonaute-2 (PDB code: 5t7b). **(D)** Sequence alignment of the VvAgo1 MID-like domain and other eukaryotic MID domains. Invariant positions are marked by an asterisk. The location that affects the allosteric regulation of miRNA binding (Djuranovic et al., 2010) is in blue. Locations involved in coordinating the two sulfate ions (Boland et al., 2010) are shown in pink.

InterproScan domain analysis was used to show the sequences identified as domains. Our results indicate the existence of an N-terminal (N) domain, a domain linker (L1), a PIWI-Argonaute-Zwille (PAZ) domain, another domain linker (L2), a P element-induced wimpy testis (PIWI) domain, and a middle (MID) domain within the VvAgo1 subfamily (Figure 3A). The domain distribution is common among the VvAgo1 subfamily (Figure 3A). Comparative analysis of the sAgos in group 2 near the VvAgo1 subfamily shown in Figure 2C indicates different types of domain distribution (Supplementary Figure S3). The specific and similar types of domain distributions represent evolutionary conservation of the VvAgo1 family, which supports the idea that purifying selection has dominated the evolution of the VvAgo1 subfamily (Supplementary Tables S5, S6).

To further characterize the domains in the VvAgo1 subfamily, VvAgo1 was selected for 3D structure modeling using SWISS-MODEL. The 3D structure of VvAgo1 displayed the conserved domains (N, PAZ, and PIWI) and two long linkers (L1 and L2) (Figure 3B). Although InterproScan domain analysis did not identify the MID domain (Figure 3A), a similar MID domain occurred in the upper-left side in the 3D structure of VvAgo1 (Figure 3B). A comparison of 3D structures showed that VvAgo1 had a predicted structure similar to human Ago, including the MID domain (Figure 3C). Moreover, the MID-like domain of VvAgo1 shares common residues in the ligand-binding sites with other eukaryotic MID domains (Figure 3D). These data confirmed the existence of the MID-like domain.

### Functional and Expression Analysis of VvAgo1

**FIGURE 4:**
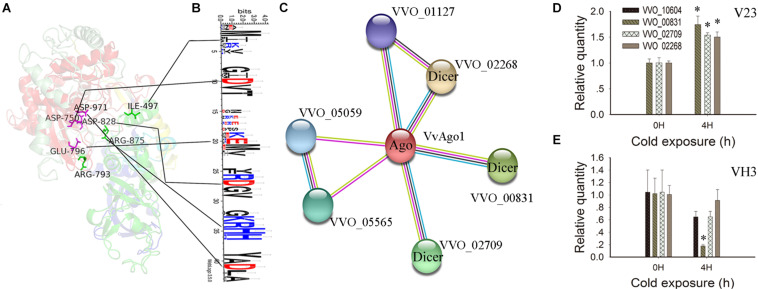
Functional and structural analysis of VvAgo1. **(A)** The 3D structure of the VvAgo1 is shown along with the potential functional motifs. The site (Ile497, Arg793, and Arg875) similar to the IRR site (Ile365, Arg635, and Arg710) participates in continuous base stacking in the hAgo2-miR-20a complex (Elkayam et al., 2012) and is displayed in green in VvAgo1. The site (Asp750, Glu796, Asp828, and Asp971) similar to the DEDD site (Asp974, Glu1013, Asp1046, and Asp1198), which might function as an RNase H-like DEDD catalytic tetrad in the *K. polysporus* Ago (Nakanishi et al., 2012), is displayed in purple in VvAgo1. **(B)** Logo analysis of motif sites in the VvAgo1 subfamily. The two upstream and downstream sites of each motif (IRR and DEDD) were used for Logo analysis. **(C)** STRING network view of VvAgo1. Colored lines between the proteins indicate various types of interaction (Szklarczyk et al., 2015). VVO_00831, VVO_002709, and VVO_02268 represent three Dicer genes according to Pfam annotation. **(D)** qPCR analysis of the expression of interacting genes in V23. **(E)** qPCR analysis of the expression of interacting genes in VH3. Bars represent the mean ± standard deviation, and one asterisk indicates *P* < 0.01 relative to 0 h.

IRR (Ile365, Arg635, and Arg710) sites participate in continuous base stacking in hAgo2-miR-20a complexes and might act as a functionally conserved switch that allows for a catalytically active conformation (Elkayam et al., 2012). A similar IRR (Ile497, Arg793, and Arg875) site was found in the VvAgo1 structure (green color, Figure 4A). WebLogo analysis showed that the Ile497 and Arg875 sites are conserved in the VvAgo1 subfamily, but not Arg793 (Figure 4B). An RNase H-like DEDD (Asp974, Glu1013, Asp1046, and Asp1198) catalytic tetrad reported in the *Kluyveromyces polysporus* Ago protein (Nakanishi et al., 2012) occurs in the VvAgo1 structure as Asp750, Glu796, Asp828, and Asp971 (shown in purple, Figure 4A). Weblogo analysis showed that Asp750, Glu796, Asp828, and Asp971 sites are conserved in the VvAgo1 subfamily (Figure 4B). Our data confirmed the existence of these putative functional sites.

Analysis of the protein-protein interactions of VvAgo1 showed that four genes were connected with VvAgo1 (VVO_10604) with four different types of interaction based on homology (Figure 4C). Pfam annotation indicated that three genes (VVO_00831, VVO_002709, and VVO_02268) belonged to the endoribonuclease Dicers, but not VVO_01127. Like Ago, Dicers are required for the biogenesis of short 5′-phosphorylated RNA guides that target complementary RNA transcripts and are also essential for the RNAi pathway. qPCR analysis confirmed the significantly upregulated expression of Dicers after cold treatment of 4 h in V23 (Figure 4D; *P* < 0.01) compared to their downregulated expression in VH3 (Figure 4E). These data suggest that CS induced a co-expression interaction network of Dicers and VvAgo1 after cold treatment in V23 for RNAi.

### Chilling Stress Triggers VvAgo1-Mediated milRNA Biogenesis

The statistics of sequences, mapping, and classification of small RNAs, as well as the detection of RNA samples, are in Supplementary Tables S7–S9 and Supplementary Figure S4. Considering that there is less evidence to support the existence of real miRNAs in fungi, these putative miRNAs and the newly predicted miRNAs were regarded as milRNAs.

**FIGURE 5:**
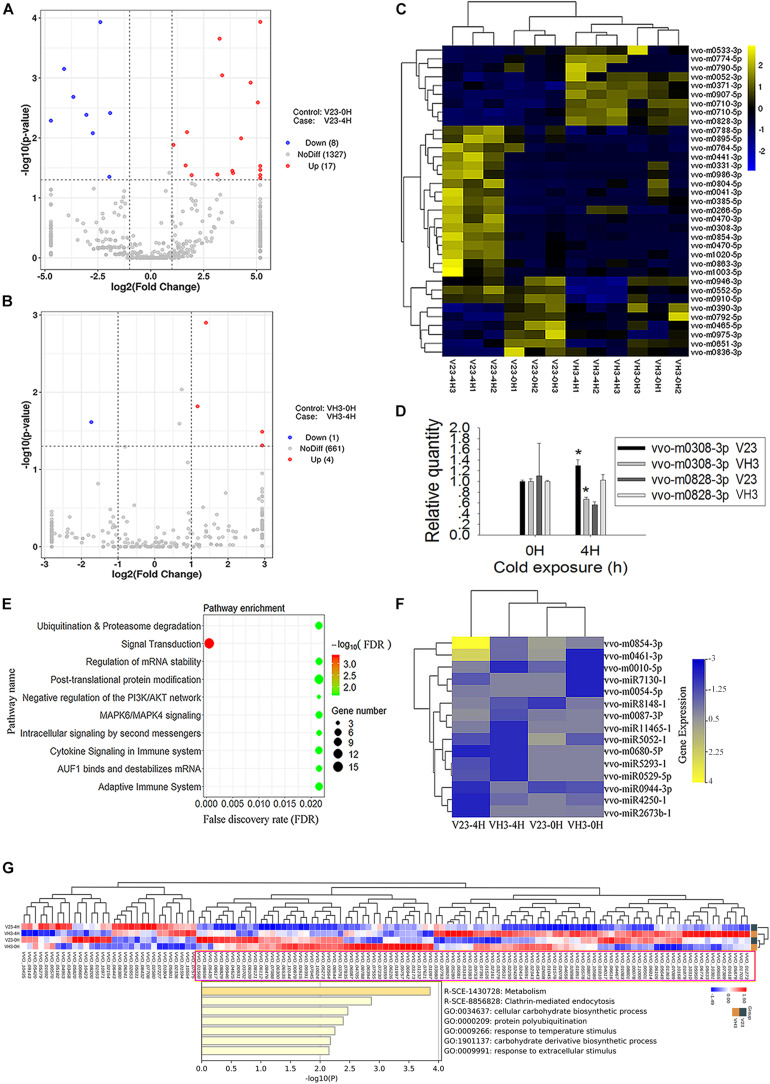
milRNA expression profile of *V. volvacea* after cold treatment. **(A)** Volcano plot of milRNA expression after cold treatment of V23 at 0 (V23-0H) and 4 h (V23-4H). **(B)** Volcano plot of milRNA expression after cold treatment of VH3 at 0 (VH3-0H) and 4 h (VH3-4H). The *x*-coordinate is the log_2_ value of the differential expressing multiples, and the *y*-coordinate is the –log_10_ *P*-value of the differential expressing significance. The vertical line in the figure represents the threshold of two times the difference. The horizontal line represents the threshold of *P*-value = 0.05. The blue dots represent low milRNA expression genes, the red dots represent high expression milRNA genes, and the gray dots represent non-significantly differentially expressed milRNA genes. **(C)** Heatmap clustering of significantly differentially expressed milRNAs. Note the lateral expression of milRNA genes, one sample per column, yellow for high milRNA expression, and blue for low milRNA expression. **(D)** qPCR analysis of the two representative milRNA expressions in V23 and VH3. The relative expression levels of indicated milRNAs were normalized to vvo-miR8786b-1. Bars represent the mean ± standard deviation, and one asterisk indicates *P* < 0.05 relative to 0 h. **(E)** Enrichment of target genes of the increased milRNA expression in V23 after cold treatment. **(F)** Heatmap of the expressed milRNAs related to ubiquitination. **(G)** Pathway and process enrichment analysis of the decreased target proteins in V23-4H. The decreased target proteins in V23-4H were obtained from the cluster marked by a purple rectangle based on the heatmap analysis of the expressed target genes of the increased milRNAs in V23.

Small RNA sequencing showed that the total number of expressed milRNA genes was 1352 in V23, higher than the 666 in VH3 (Figures 5A,B). Analysis of expressed genes showed 17 significantly upregulated milRNA genes and 8 significantly downregulated genes (Figure 5A and Supplementary Table S10), while the total number of obviously expressed milRNA genes was only 5 in VH3 (Figure 5B and Supplementary Table S11). A heatmap of significantly expressed milRNAs showed that CS induced increased milRNA expression in V23, which did not occur in VH3 (Figure 5C). The two representative milRNAs (vvo-m0308-3p and vvo-m0828-3p) had the highest and lowest log_2_ fold-change values after CS for 4 h in V23, respectively (Supplementary Table S10). The secondary structures of the two folded precursors are shown in Supplementary Figure S5. CS did not trigger the expression of the conserved vvo-miR8786b-1 (Supplementary Tables S10, S11), so it was used as a reference to normalize the expression of target milRNAs. qPCR analysis showed that the SEMs of the C_t_ values of vvo-miR8786b-1 in V23 and VH3 were only 0.0189 and 0.110, respectively, confirming its stable expression after CST. The qPCR results of two representative milRNAs showed expression trends similar to small RNA sequencing results (Figure 5D and Supplementary Tables S10, S11). For example, the qPCR analysis showed that CS induced the significantly upregulated expression of vvo-m0308-3p in V23 compared to its significantly downregulated expression in VH3 (Figure 5D). Together with the co-expression network of Dicers and VvAgo1 (Figures 1C, 4D), the increased milRNA expression suggested that CS triggered VvAgo1-mediated milRNA biogenesis in V23.

The predicted target genes of the increased milRNA in V23 were enriched in multiple pathways such as signal transduction, ubiquitination, and proteasome degradation (Figure 5E and Supplementary Table S12). Heatmap analysis showed that CS altered the expression profile of milRNAs with their predicted target genes related to ubiquitination (Figure 5F) and signal transduction in V23 (Supplementary Figure S6), but this change did not occur in VH3. Combined analysis of transcriptome and proteome data confirmed that most of the target genes of expressed milRNAs (Supplementary Figure S7A) were not translated into proteins (Supplementary Figure S7B). Heatmap analysis revealed the decreased target proteins of the increased milRNAs in V23-4H (Figure 5G). Pathway and process enrichment analysis of the reduced target proteins in V23-4H were enriched in the pathway and processes, such as metabolism, clathrin-mediated endocytosis, protein polyubiquitination, and response to temperature stimulus, which are essential for responses to environmental stimuli (Figure 5G). These data suggested that VvAgo1-mediated RNAi facilitated the cryogenic autolysis of *V. volvacea* by milRNA.

## Discussion

RNA silencing is a sequence-specific gene regulation system conserved in eukaryotes, and the Ago protein family is at its core. The gene silencing mechanisms mediated by small nucleic acids in which Ago proteins are vital players exist in all domains of life, from bacteria to eukaryotes (Miyoshi et al., 2016). Phylogenetic analysis of eukaryotic Agos suggests that the two sAgo lineages have diverged after ancient gene duplication and have had an independent evolutionary trajectory (Figure 2A). The evolution of the sAgo lineages has featured additional gene duplications and subsequent gene loss (Figure 2C), which partially supports the view that additional species-specific duplications and losses of more recent origin have happened after an ancient duplication of basidiomycetes Ago genes (Hu et al., 2013).

Based on both their phylogenetic relationships and their capacity to bind to small RNAs, Ago proteins can be classified into three groups (Meng et al., 2013): (I) Ago-like subfamily members that bind to miRNAs and small interfering RNAs (siRNAs); (II) the PIWI-like subfamily, members of which bind to PIWI-interacting RNAs (piRNAs); and (III) the WAGO subfamily (worm-specific Argonautes) that bind to secondary siRNAs (Yigit et al., 2006; Tolia and Joshua-Tor, 2007; Hutvagner and Simard, 2008). Evolutionary analysis indicated that VvAgo1 belongs to the Ago-like subfamily, and its evolution has consisted of gene duplication, subsequent gene loss, and purifying selection (Supplementary Tables S5, S6).

Our structural and sequence analysis suggested that VvAgo1 encodes a functional Ago protein (Figures 3, 4). The crystal structures of fungal Agos were first identified in *K. polysporus* (Nakanishi et al., 2012). Like other Agos (Hutvagner and Simard, 2008; Elkayam et al., 2012), *K. polysporus* Ago features an N domain, a PAZ domain, a MID domain, and a PIWI domain, along with two domain linkers, L1 and L2 (Nakanishi et al., 2012). However, domain analysis of the VvAgo1 subfamily showed N, L1, PAZ, L2, and PIWI domains but not a MID domain (Figure 3A and Supplementary Figure S3). A comparison of the 3D structures and ligand-binding sites do, however, indicate the existence of a MID-like domain in *V. volvacea* (Figures 3B–D). Functional motif analysis has shown that the VvAgo1 subfamily has incomplete IRR sites compared to reported human Ago (Elkayam et al., 2012) and the complete catalytic tetrad reported in *K. polysporus* Ago (Nakanishi et al., 2012). Together with the similar distribution of domains in the VvAgo1 subfamily (Figure 3A), these data indicate the specificity of this subfamily.

Here, our observations suggested that VvAgo-mediated RNAi plays a vital role in facilitating the cryogenic autolysis of *V. volvacea*. Ago protein is a critical player in eukaryotic RNAi pathways in which Ago utilizes short 5′-phosphorylated RNA guides to target complementary RNA transcripts (Swarts et al., 2014). Ago proteins bind small RNAs and use their sequence information to guide the silencing of their target RNAs (Sasaki and Tomari, 2012). Like Ago, Dicer is also a key enzyme involved in RNAi. It is required for the biogenesis of miRNAs and siRNAs and plays an essential role in the effector step of RNA silencing, RNA-induced silencing complex (RISC) assembly (Jaskiewicz and Filipowicz, 2008). RISC has Ago as a catalytic component, which is an endonuclease capable of degrading mRNA. Therefore, Dicer and Ago proteins consist of the core parts of the RNAi pathway. The upregulated expression network of Dicers and VvAgo1 after cold treatment in V23 but not in VH3 (Figures 1C, 4D) suggests that CS triggers VvAgo1-mediated milRNA biogenesis. Small RNA sequencing and qPCR analysis confirmed that CS triggered the increased milRNA expression in V23 (Figures 5A,D).

The predicted target genes of the increased milRNA in V23 after cold treatment were enriched in multiple pathways such as signal transduction, ubiquitination and proteasome degradation, and post-translational protein modification (Figure 5E). The increase in milRNAs could lead to mRNA target gene degradation and the subsequent inhibition of their mediated cellular processes. For example, increased milRNAs related to ubiquitination could result in the degradation of target genes in the ubiquitination pathway (Figure 5G) and thus weaken it. Weakened ubiquitination in turn could affect normal cellular processes such as signal transduction, proteasome degradation, and post-translational protein modification, finally facilitating cryogenic autolysis. Previous studies have reported that ubiquitination is involved in cryogenic autolysis (Gong et al., 2015, 2016). Our observations suggest that VvAgo1-mediated RNAi might facilitate the cryogenic autolysis of *V. volvacea* by affecting ubiquitination.

The specific and similar types of domains distributed in the VvAgo1 family (Figure 3A) represent evolutionary conservation, which suggests that they have a similar function. The functional role of VvAgo1 in cryogenic autolysis may be due to the VvAgo1-mediated RNAi pathway in V23. Some fungi might have evolved different VvAgo1-mediated RNAi pathways for cold stress response, although they have similar types of VvAgo1. This view is consistent with RNA silencing pathways in fungi having diversified significantly during evolution in response to environmental conditions (Nakayashiki et al., 2006). Several studies reported the roles of miRNAs in response to CS in plants and animals. For example, genome-wide identification of chilling responsive miRNAs was reported in *Prunus persica* (Barakat et al., 2012) and the regulatory role of miRNAs in gene plasticity during cold shock was discovered in zebrafish larvae (Hung et al., 2016). However, few studies have investigated the role of milRNAs in response to CS in fungi. Our observations showed that CS induced the increased milRNAs with their predicted target genes related to the signal transduction in V23 instead of VH3 (Supplementary Figure S6). Together with the decreased target proteins in V23-4H that were enriched in the pathway associated with environmental stimuli (Figure 5G), these data suggest that VvAgo1-mediated RNAi facilitated the cryogenic autolysis of *V. volvacea* by milRNAs by affecting signal transduction.

Recent findings in the regulation of gene expression in response to their environment by small RNAs in fungi have led to the understanding of the regulatory processes better to survive environmental stresses, which also represents an enormous potential for application in the fields of medicine and agriculture (Villalobos-Escobedo et al., 2016). The related genes in the VvAgo1-mediated RNAi pathways have the potential to be the new target genes for reducing cryogenic autolysis. Together with the recently reported degradation technology of fungal milRNAs (Wang et al., 2019), our identified milRNAs could also be applied for inhibitor compound or target genes for regulating cryogenic autolysis in *V. volvacea*. These observations provide technical and theoretical guidance for further research and development of low temperature preservation technology of *V. volvacea.*

## Conclusion

Our results confirmed that VvAgo1 was upregulated in response to CS. VvAgo1 belongs to the Ago-like family, and during its evolution, it has undergone gene duplication, subsequent gene loss, and purifying selection. VvAgo1 encodes a functional Ago protein, and CS might trigger VvAgo1-mediated RNAi pathways to facilitate the cryogenic autolysis of *V. volvacea*.

## Data Availability Statement

The datasets generated for this study can be found in the NCBI under the accession numbers of PRJNA594834.

## Author Contributions

MG and DB designed the research. YW, YZ, JW, JS, YyW, and YL performed the research. MG, JZ, QT, and RY analyzed the data. MG wrote the manuscript. All the authors contributed to the article and approved the submitted version.

## Conflict of Interest

The authors declare that the research was conducted in the absence of any commercial or financial relationships that could be construed as a potential conflict of interest.

## Footnotes

1 http://www.ebi.ac.uk/interpro/

2 http://www.datamonkey.org/

3 https://topmatch.services.came.sbg.ac.at/

## Supplementary Material

FIGURE S1 qPCR analysis of the four Agos expressed in V23. Bars represent the mean ± standard deviation, and one asterisk indicates *P* < 0.01 relative to 0 h.

FIGURE S2 Phylogenetic analysis of eukaryotic Agos. A total of 131 eukaryotic Ago proteins were used for multiple sequence alignment of conserved blocks of MID and PIWI domains.

FIGURE S3 Functional domain analysis of the sAgos in group 2 in Figure 2C.

FIGURE S4 Detection of RNA samples in V23 and VH3 after cold treatment.

FIGURE S5 Secondary hairpin structures of the two folded precursors from *V. volvacea*. The representative milRNAs (vvo-m0308-3p and vvo-m0828-3p) are marked in red and the reads are listed.

FIGURE S6 Heatmap of the expressed milRNAs related to signal transduction.

FIGURE S7 Combined analysis of the target genes of expressed milRNAs. **(A)** Heatmap of the target mRNAs of expressed milRNAs. **(B)** Heatmap of the target proteins of expressed milRNAs.

TABLE S1 Primers used for qPCR.

TABLE S2 CLUSTAL alignment between VvAgo1 and the template of human Argonaute-2 (PDB code: 5t7b).

TABLE S3 Five Ago genes were identified in the *V. volvacea* genome using InterProScan.

TABLE S4 Five Ago genes identified in *V. volvacea* and their expression at 2 and 4 h in V23 or VH3. Their expression values are based on high-throughput sequencing analysis of mRNAs (Bao et al., 2013; Gong et al., 2020).

TABLE S5 Detection of natural selection in the VvAgo1 subfamily. The probability of rejecting the null hypothesis of strict neutrality (dN = dS) in favor of the alternative hypothesis (dN > dS) (below diagonal) is shown. *P*-values below 0.05 are considered significant at the 5% level and are highlighted. The test statistic (dN–dS) is shown above the diagonal.

TABLE S6 Selection pressure analysis of the VvAgo1 subfamily using SLAC, FEL, and IFEL methods. (www.datamonkey.org).

TABLE S7 Sequence statistics of samples.

TABLE S8 Mapping statistics of samples.

TABLE S9 Classification statistics of small RNAs (total).

TABLE S10 Expressed milRNAs in V23 after cold treatment for 0 and 4 h. Colors represent significantly differently expressed milRNAs.

TABLE S11 Expressed milRNAs in VH3 after cold treatment for 0 and 4 h. Colors represent significantly differently expressed milRNAs.

TABLE S12 Target genes of total significantly differently expressed milRNAs.

TABLE S13 Primers used for qPCR of milRNAs.
