# Supplementary figures and images for: Chilling Stress Triggers VvAgo1-Mediated miRNA-Like RNA Biogenesis in Volvariella volvacea
Source: Front Microbiol. 2020 Sep 15;11:523593. doi: 10.3389/fmicb.2020.523593 (PMC7522536; doi:10.3389/fmicb.2020.523593)

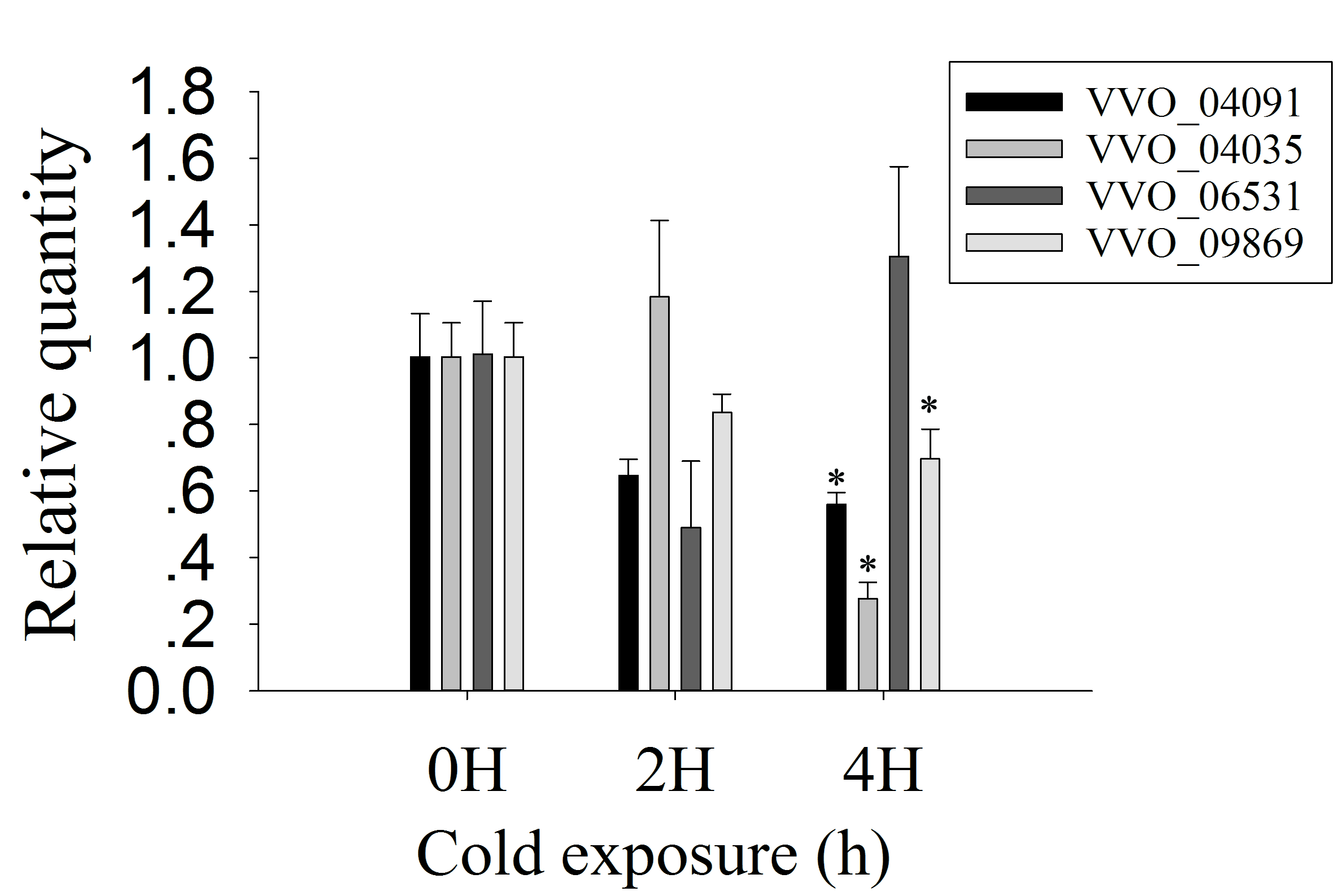

Supplement: FIGURE S1 — qPCR analysis of the four Agos expressed in V23. Bars represent the mean ± standard deviation, and one asterisk indicates P < 0.01 relative to 0 h. [file Image_1.TIF]

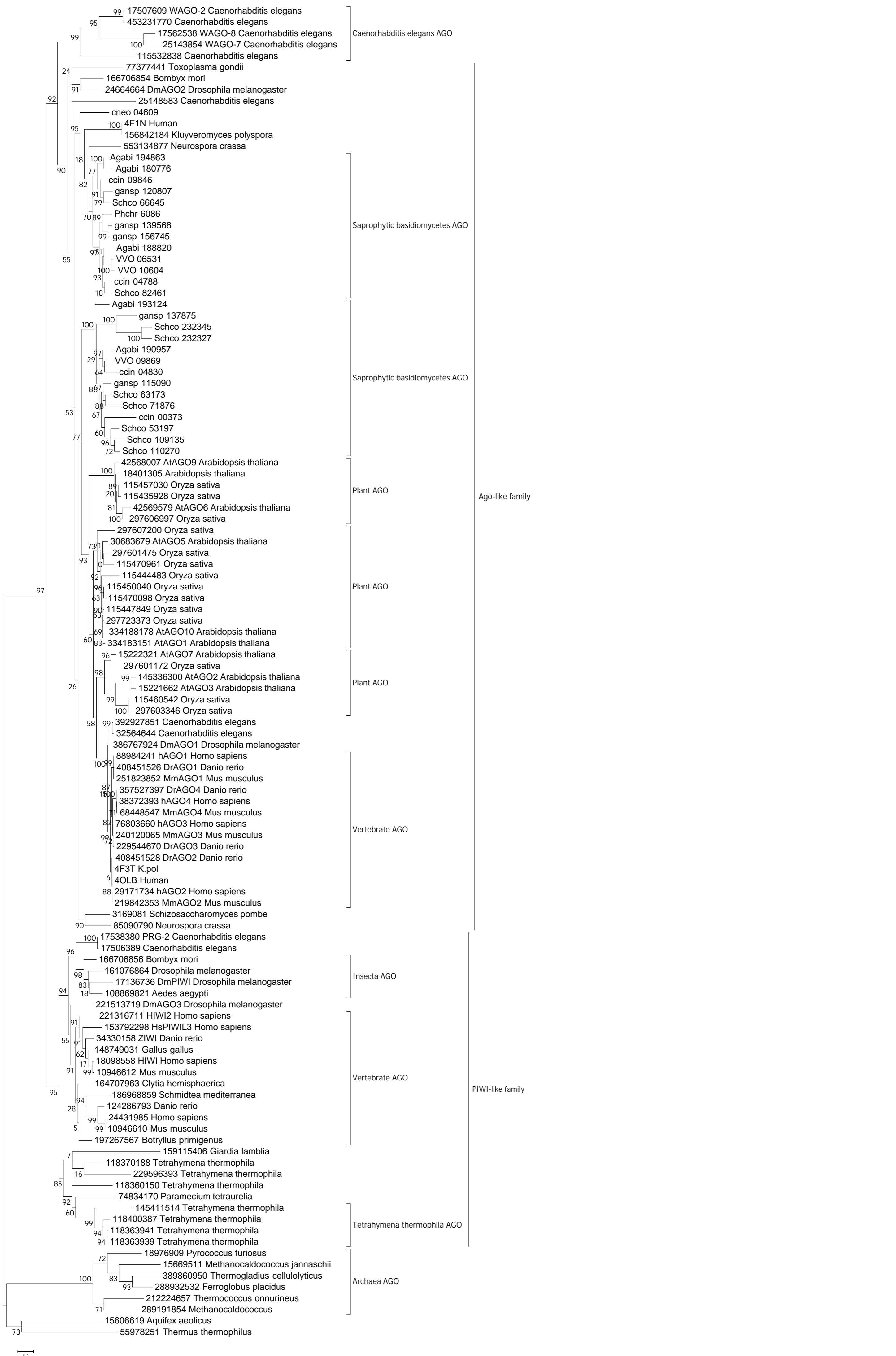

Supplement: FIGURE S2 — Phylogenetic analysis of eukaryotic Agos. A total of 131 eukaryotic Ago proteins were used for multiple sequence alignment of conserved blocks of MID and PIWI domains. [file Image_2.PDF]

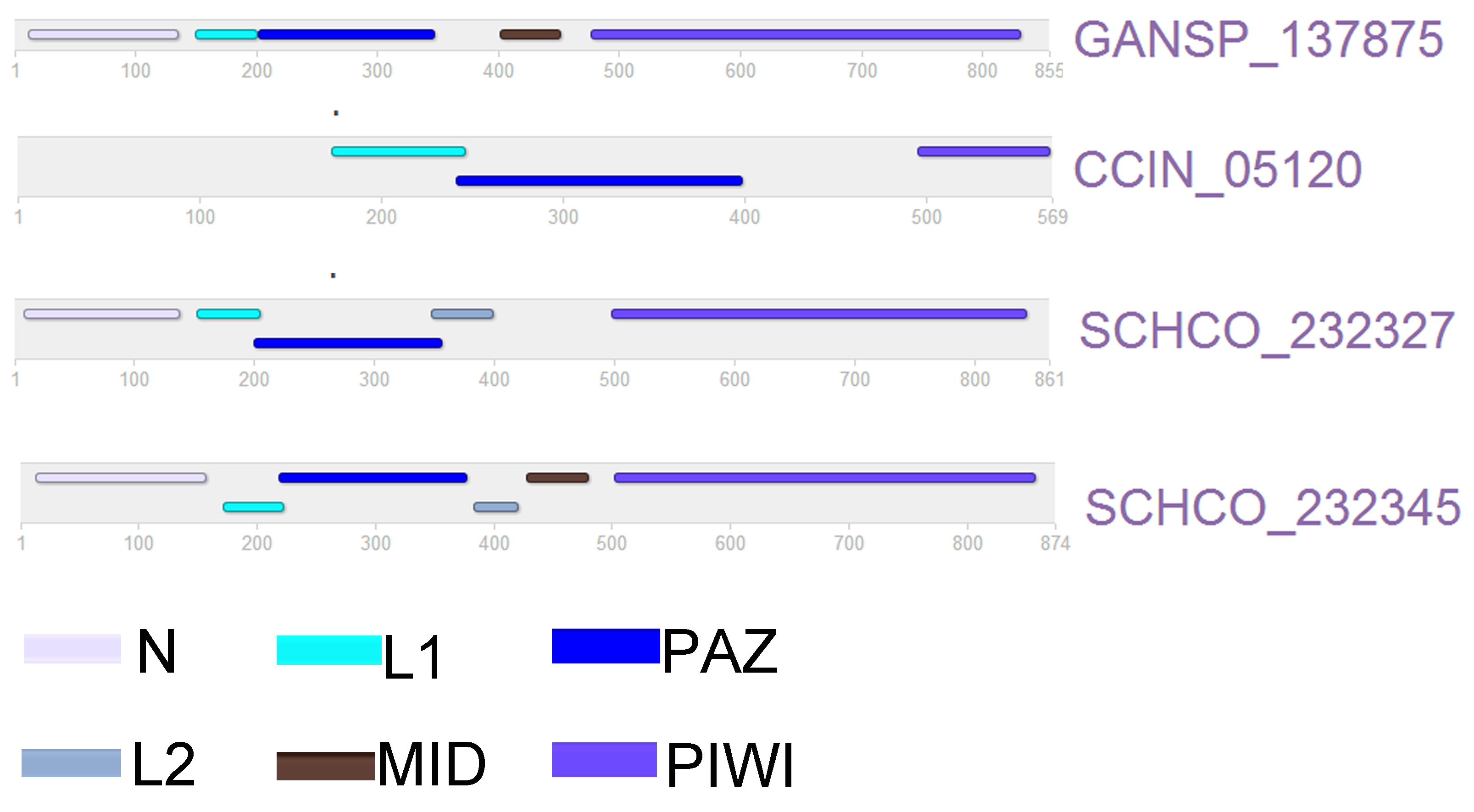

Supplement: FIGURE S3 — Functional domain analysis of the sAgos in group 2 in Figure 2C. [file Image_3.TIF]

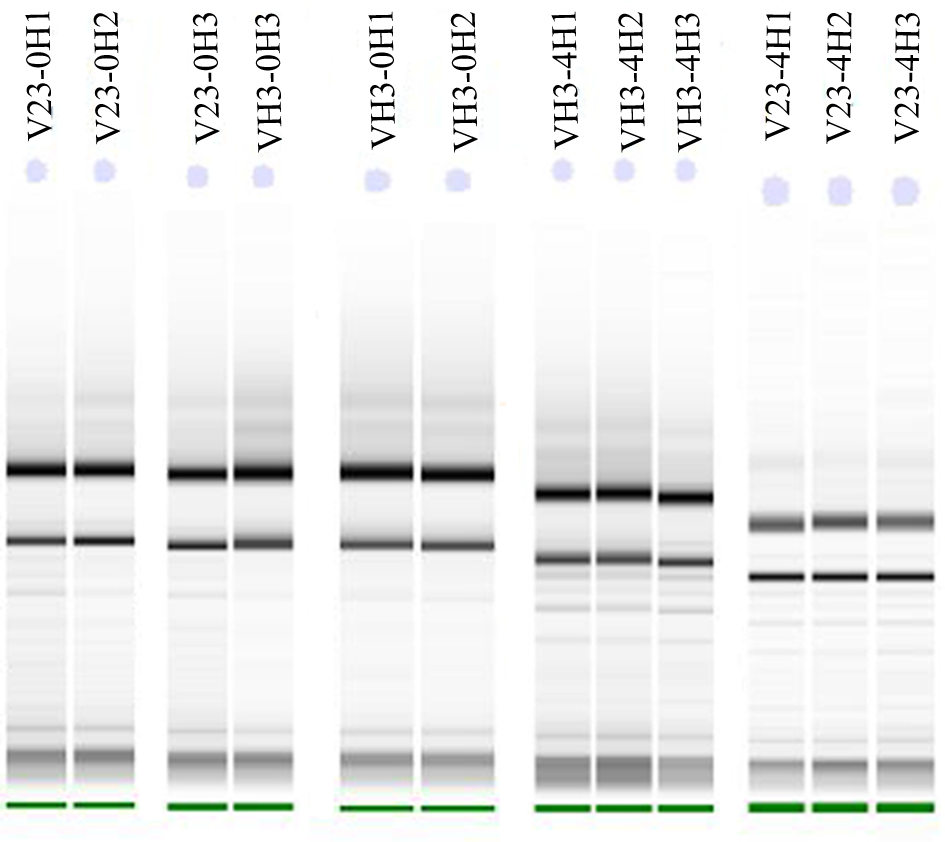

Supplement: FIGURE S4 — Detection of RNA samples in V23 and VH3 after cold treatment. [file Image_4.TIFF]

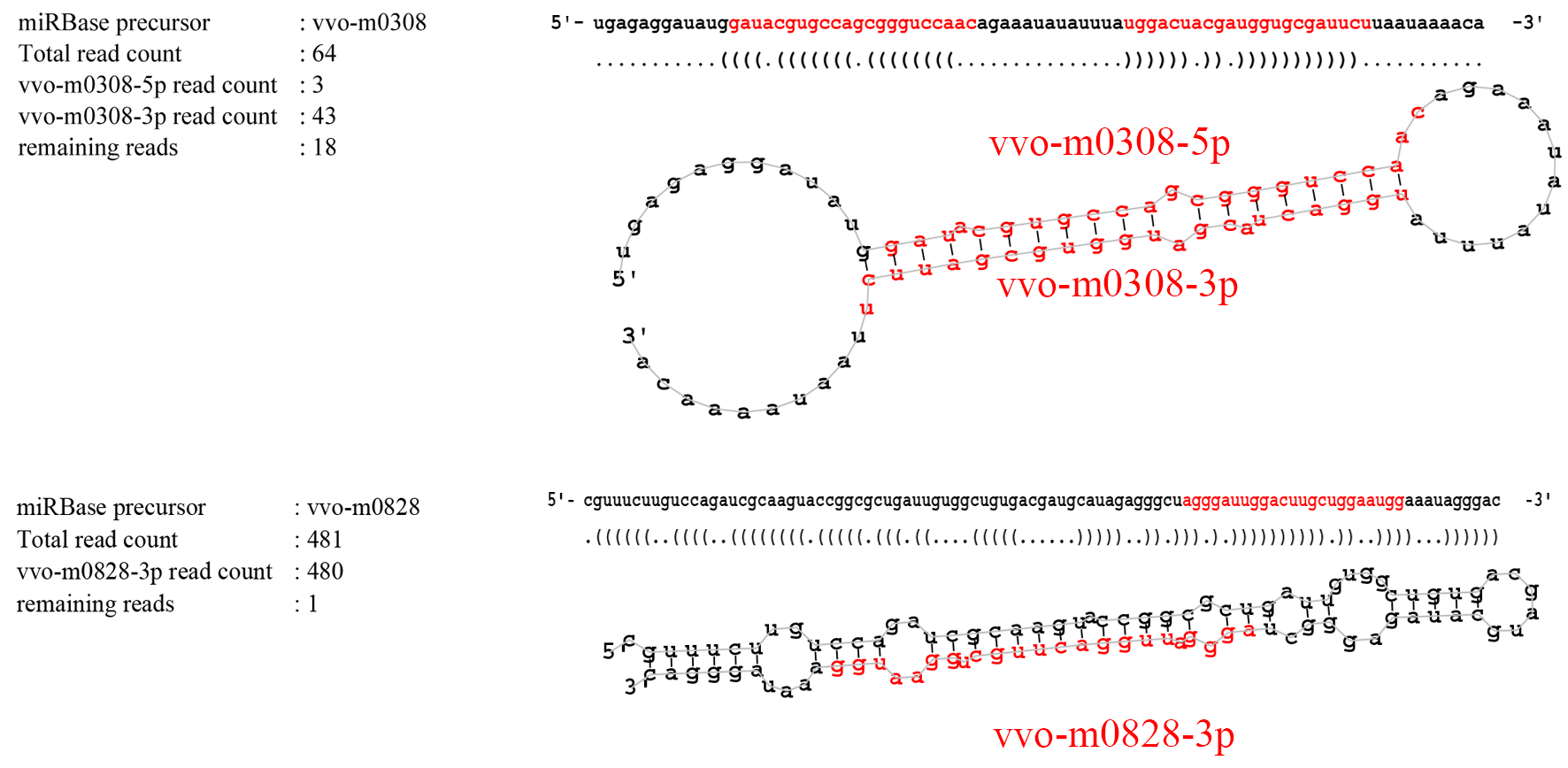

Supplement: FIGURE S5 — Secondary hairpin structures of the two folded precursors from V. volvacea. The representative milRNAs (vvo-m0308-3p and vvo-m0828-3p) are marked in red and the reads are listed. [file Image_5.TIFF]

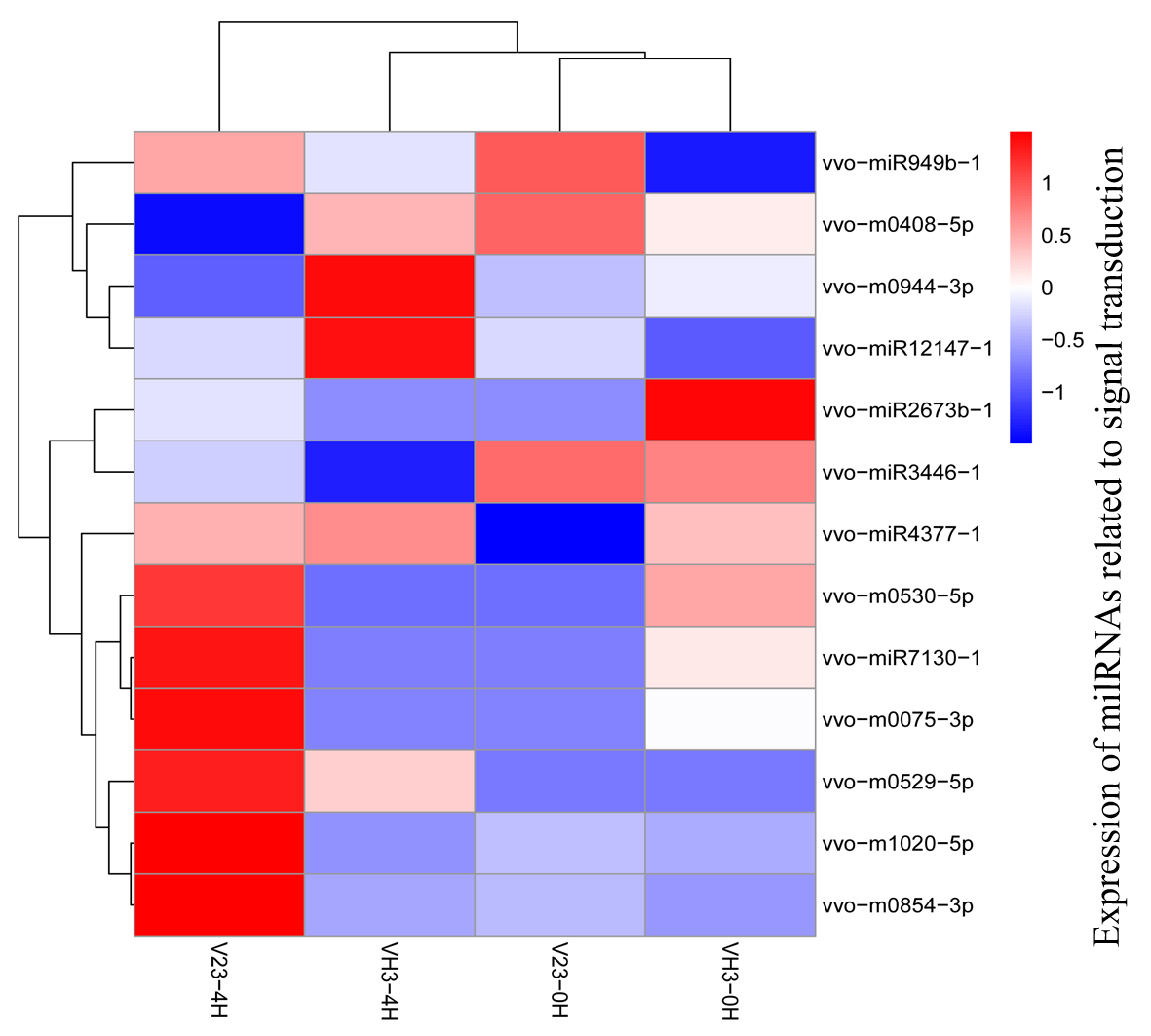

Supplement: FIGURE S6 — Heatmap of the expressed milRNAs related to signal transduction. [file Image_6.TIFF]

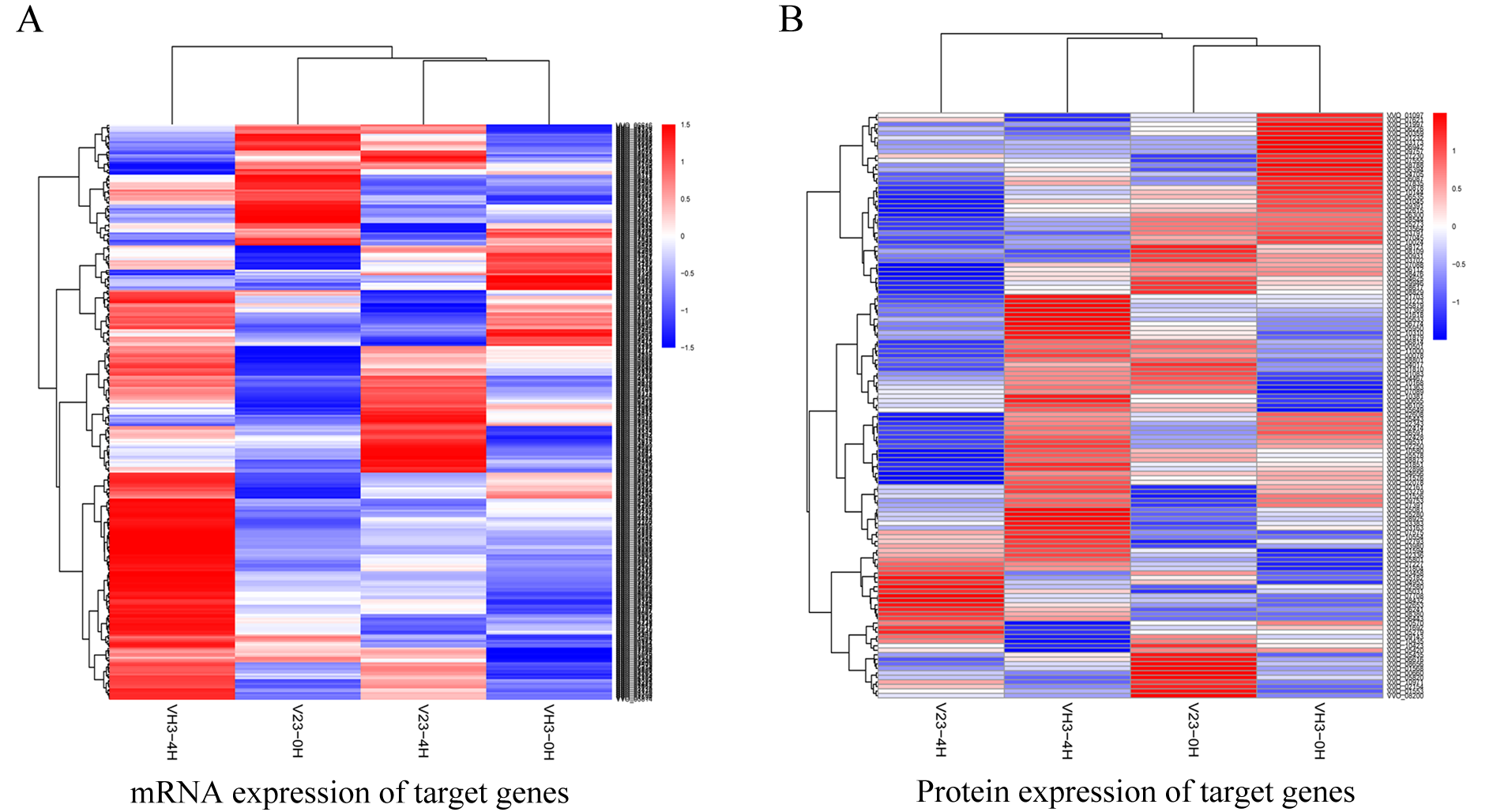

Supplement: FIGURE S7 — Combined analysis of the target genes of expressed milRNAs. (A) Heatmap of the target mRNAs of expressed milRNAs. (B) Heatmap of the target proteins of expressed milRNAs. [file Image_7.TIFF]
