# Supplementary material for: Chilling Stress Triggers VvAgo1-Mediated miRNA-Like RNA Biogenesis in Volvariella volvacea
Source: Front Microbiol. 2020 Sep 15;11:523593. doi: 10.3389/fmicb.2020.523593 (PMC7522536; doi:10.3389/fmicb.2020.523593)
Supplement: TABLE S1 — Primers used for qPCR. [file Data_Sheet_1.zip › Table 1.docx]

Table S1 Primers used for qPCR

| Gene | primer |
| --- | --- |
| *GAPDH*-F | CCTCTGGCCAAAGTCATCCA |
| *GAPDH*-R | AGGGCCCATCGACAGTCTT |
| *VvAgo1*-F | TGGGCATCCACACGGTTAT |
| *VvAgo1*-R | CCAAGTTTCGCGTTGACCTT |
| *VVO_04091-*F | CCCAATCCTCAAGGCTCTCG |
| *VVO_04091-*R | CCTTGCAATCCGTCTCCGAT |
| *VVO_04305-*F | GCACAACTCGTCCAACACAC |
| *VVO_04305-*R | TTCCACAAGGTCTCGGCTTC |
| *VVO_06531-*F | CACACGACTACACCCAACGA |
| *VVO_06531-*R | CGTATCGGCATTTGGCTTGG |
| *VVO_09869-*F | TTGCAGGATGACAATCCCTT |
| *VVO_09869-R* | GATTGGTGAAGGTTGAACGC |
| *VVO_00831*-F | GCAACACCTCCCACAAAACC |
| *VVO_00831*-R | TCGTAATGCTATGACCCCGC |
| *VVO_01127* -F | CGATCTTCCCAAACCGCTCT |
| *VVO_01127* -R | CGGTGGTGTGGTCGTAATCT |
| *VVO_02268* -F | CCGCCTATATCCGACGCTTT |
| *VVO_02268* -R | TACACGCTCACACCCAACTC |
| *VVO_02709*-F | GGGCCACTCTTACACTCCAC |
| *VVO_02709*-R | GCGGTATTCCTCGGGTACAG |
