# Supplementary material for: Chilling Stress Triggers VvAgo1-Mediated miRNA-Like RNA Biogenesis in Volvariella volvacea
Source: Front Microbiol. 2020 Sep 15;11:523593. doi: 10.3389/fmicb.2020.523593 (PMC7522536; doi:10.3389/fmicb.2020.523593)
Supplement: TABLE S1 — Primers used for qPCR. [file Data_Sheet_1.zip › Table 2.docx]

Table S2 CLUSTAL alignment between VvAgo1 and the template of human Argonaute-2 (PDB code: 5t7b)

_______________________________________________________________________________

Target -------------MSSDNAPSRNSGRGRGRGRGRRSNARGRGRGASSEGGSGINPHDTGP

5t7b.1.B ------------------------------------------------------------

Target VSSESSRGGSAPRGGFRGNSRGRGRGGSRNRRGAGDVGGGPRVVDKGIFNEGVPAVVPSH

5t7b.1.B -------------------------------------GAGP------------------A

*.**

Target LSNDKLSSLIQSYTTFPHS-----DRPLRPDFGTKGAASLVRANFFAVQH-EGSIFDYVV

5t7b.1.B LAPPAPPPPIQGYAF---------KPPPRPDFGTSGRTIKLQANFFEMDIPKIDIYHYEL

*: .. **.*: . * ******.* : ::**** :: : .*:.* :

Target TITPTNSTQESSSSTPKSVPNAKKKRLFELLEELFEQDSSLAWCLPHIAHDYAQRMVSSQ

5t7b.1.B DIKPEKC------------PRRVNREIVEHMVQHFKTQIF---GDRKPVFDGRKNLYTAM

*.* :. *. ::.:.* : : *: : : ..* :.: ::

Target ELPQ---PLEVTITYRDEGEEEPRPNADVYLISIKFERELESEDSKSYLEGRPDKRDFDI

5t7b.1.B PLPIGRDKVELEVTLPGE------GKDRIFKVSIKWVSCVSLQALHDALSGRLPSVP---

** :*: :* .* : :: :***: :. : :. *.** .

Target QPYVSAINLFLQQFAGRTGVRVGKNRYFFQSMTESRSFELNIGLEAWKGFSISTRPSYGQ

5t7b.1.B FETIQALDVVMRHLPSMRYTPVGRS--FFTA-SEGCSNPLGGGREVWFGFHQSVRPSLWK

:.*:::.::::.. . **:. ** : :*. * *. * *.* ** *.*** :

Target LLVNIHVCWAAFVRPGNLAQRLQEFRDSS--------E----GGMPSRMVNSIKVKLTHL

5t7b.1.B MMLNIDVSATAFYKAQPVIEFVCEVLDFKSIEEQQKPLTDSQRVKFTKEIKGLKVEITHC

:::**.*. :** :. : : : *. * . :: ::.:**::**

Target G---YTKRLFSIGSKAPRNIKFQC-EEFG---EISVEEYFKKSDFIFFAFFPFLHFRTIL

5t7b.1.B GQMKRKYRVCNVTRRPASHQTFPLQQESGQTVECTVAQYFKD------------------

* . *: .: :.. : .* :* * * :* :***.

Target IMIFISAGYDRELKHPDLPVVNVGNRAKAIWVPPELCEILPGCAYRGKLDEYETSMMIRH

5t7b.1.B -------RHKLVLRYPHLPCLQVGQEQKHTYLPLEVCNIVAGQRCIKKLTDNQTSTMIRA

:. *::*.** ::**:. * ::* *:*:*:.* ** : :** ***

Target ACNPPKTNFESIVDDGLPTLGFKRDEDTSDSLEGFGLSVDLDPIVIPSRELPPPRISYQG

5t7b.1.B TARSAPDRQEEI-SKLMRSASFNTD----PYVREFGIMVKDEMTDVTGRVLQPPSILYGG

:.... . *.* .. : : .*: * :. **: *. : :..* * ** * * *

Target S---TPQIQNGGWNIVNAKFHRSATVNSWSVLVVQDSRNPFIAGLEDQKLTVIVDGFKEK

5t7b.1.B RNKAIATPVQGVWDMRNKQFHTGIEIKVWAIACFAPQRQ-----CTEVHLKSFTEQLRKI

. :* *:: * :** . :: *:: . .*: : :*. :.: :::

Target CASTGMTMPEAAPRTEIVQLLPLKGDEGRSNSIEILRRKLGEQLSTGDRPDFVLVLLEKT

5t7b.1.B SRDAGMPIQG-QPCF-----CKY--A-QGADSVEPMFRHLKNTYA---GLQLVVVILPGK

. .:**.: * ::*:* : *:* : : ::*:*:* .

Target DRFIYPALKCIGDVKLGIHTVIMQARTVMRGKRDQYFSNVALKVNAKLGGVNHRLDDGAM

5t7b.1.B -TPVYAEVKRVGDTVLGMATQCVQMKNVQ-RTTPQTLSNLCLKINVKLGGVNNILLPQGR

:*. :* :**. **: * :* :.* . * :**:.**:*.******: * .

Target TWLTKKKTMLVGIDVTHRSPGSQEGTPSIAAMVANVDDSFVQFPASIKLQTSERGERPNE

5t7b.1.B PPVFQQPVIFLGANVTHPPAGDG-KKPSIAAVVGSMDAHPNRYCATVRVQQHR-----QE

. : :: .:::* :*** ..*. .*****:*..:* :: *::::* . :*

Target MVDDIEVMLVERLGLYQERNGVLPERIFVFRDGVSEGQFDIVLEEELKGIRKAFKNPKFQ

5t7b.1.B IIQDLAAMVRELLIQFYKSTRFKPTRIIFYRNGVSEGQFQQVLHHELLAIREACIKLEK-

:::*: .*: * * : : . . * **:.:*:*******: **..** .**:* : :

Target NPDGSAYRPLLSIIICGKRHHARMAPTNAQF-KDKGGNTRPGTVIDKGITAVYDFDFYLQ

5t7b.1.B -----DYQPGITFIVVQKRHHTRLFCTDKNERVGKSGNIPAGTTVDTKITHPTEFDFYLC

*:* :::*: ****:*: *: : .*.** .**.:*. ** :*****

Target SHVAIQGHAKGTHYTVVYDENRFTADEIQQGIHNMCHLYARATRAVSLVPPAYYADLACE

5t7b.1.B SHAGIQGTSRPSHYHVLWDDNRFSSDELQILTYQLCHTYVRCTRSVSIPAPAYYAHLVAF

**..*** :: :** *::*:***::**:* :::** *.*.**:**: .*****.*..

Target RARFYLNEFFDAAVETASSIASGSKGKGKKRDK-QEERESVFREAVKAWGEGLHDDIKNT

5t7b.1.B RARYHLVDKEHDSAEGS-------HTSG------QSNGRDHQALA-KA--VQVHQDTLRT

***::* : . :.* : : .* *.: .. * ** :*:* .*

Target MFYQ

5t7b.1.B MYFA

*:: ______________________________________________________________________________

*Note:* The marks “*”stand for the identical amino acids.
