# Supplementary material for: Chilling Stress Triggers VvAgo1-Mediated miRNA-Like RNA Biogenesis in Volvariella volvacea
Source: Front Microbiol. 2020 Sep 15;11:523593. doi: 10.3389/fmicb.2020.523593 (PMC7522536; doi:10.3389/fmicb.2020.523593)
Supplement: TABLE S1 — Primers used for qPCR. [file Data_Sheet_1.zip › Table 3.doc]

**Table S3 Five Ago genes were identified in the *V. volvacea* genome using InterProScan**

>VVO_10604, Argonaute protein, PAZ, IPR003100

MSSDNAPSRNSGRGRGRGRGRRSNARGRGRGASSEGGSGINPHDTGPVSSESSRGGSAPRGGFRGNSRGRGRGGSRNRRGAGDVGGGPRVVDKGIFNEGVPAVVPSHLSNDKLSSLIQSYTTFPHSDRPLRPDFGTKGAASLVRANFFAVQHEGSIFDYVVTITPTNSTQESSSSTPKSVPNAKKKRLFELLEELFEQDSSLAWCLPHIAHDYAQRMVSSQELPQPLEVTITYRDEGEEEPRPNADVYLISIKFERELESEDSKSYLEGRPDKRDFDIQPYVSAINLFLQQFAGRTGVRVGKNRYFFQSMTESRSFELNIGLEAWKGFSISTRPSYGQLLVNIHVCWAAFVRPGNLAQRLQEFRDSSEGGMPSRMVNSIKVKLTHLGYTKRLFSIGSKAPRNIKFQCEEFGEISVEEYFKKSDFIFFAFFPFLHFRTILIMIFISAGYDRELKHPDLPVVNVGNRAKAIWVPPELCEILPGCAYRGKLDEYETSMMIRHACNPPKTNFESIVDDGLPTLGFKRDEDTSDSLEGFGLSVDLDPIVIPSRELPPPRISYQGSTPQIQNGGWNIVNAKFHRSATVNSWSVLVVQDSRNPFIAGLEDQKLTVIVDGFKEKCASTGMTMPEAAPRTEIVQLLPLKGDEGRSNSIEILRRKLGEQLSTGDRPDFVLVLLEKTDRFIYPALKCIGDVKLGIHTVIMQARTVMRGKRDQYFSNVALKVNAKLGGVNHRLDDGAMTWLTKKKTMLVGIDVTHRSPGSQEGTPSIAAMVANVDDSFVQFPASIKLQTSERGERPNEMVDDIEVMLVERLGLYQERNGVLPERIFVFRDGVSEGQFDIVLEEELKGIRKAFKNPKFQNPDGSAYRPLLSIIICGKRHHARMAPTNAQFKDKGGNTRPGTVIDKGITAVYDFDFYLQSHVAIQGHAKGTHYTVVYDENRFTADEIQQGIHNMCHLYARATRAVSLVPPAYYADLACERARFYLNEFFDAAVETASSIASGSKGKGKKRDKQEERESVFREAVKAWGEGLHDDIKNTMFYQ

>VVO_06531, Argonaute protein, PAZ, IPR003100

MSSNRGRGSGRGRGSAGPPPGGSGSTHPHNTSPAPSGPSRGGFAPRGGFRGGDRGGRGYGGSRGRGGGGNVGGGHRDGSVIFNEGAPAAVPSHLSNDKLGALIRSYNSVPHRPHRPLRPGFGSAGAPSVVRANFFAVKHEGPIYDYTVTITQSSGKGSGSKSSAATGVANAKKRRLFKLLEQEGAIKPYITNIAHDYTQRLVSSKQLPQPLDLDITYYDEDEDKPKPNADTYLVSIKFERELKPEDSKSYLEGSLDKRDFDIQPYASATNLFLQQLASRTGVRVGKNRYFFQQLSQSLRLSTGIEAWKGFSISTRPSYGQLLVNVNVCWAAFVVPGNLADALNAFNANSRGAMPTLPKQMVSSIKVKLRHLGYHKKLFAIGTKTPRDTYFDCAEFGGQISVEQYFKKKYNIQLKFPADLPVVDVGTRAKTIWVPPELCDILPGCAYRGKLNDRETAQMIRYACNDPRTNFESIVNEGLPILGFKKIAGSNAPDSLAGFKLSVDPNPIVIPSRELPPPRISYKQGRADIRNGGWNILNVKFHRGARVNSWWVLVVQDSPNPSISSPDDPILVGLVNGFKNKCISSGMTMPAANPRTFSVQLVNPRRDPGRAESIRKLRELLTRQVNEAGRPDFVLVILENRDNFIYPALKRLGDVDLGVHTVHMQIDKATGDPRKQDQYFSNVALKVNAKLGGVNHLLDPSAMTWLTKKKTMLVGIDVTHRGPGSREGTPSIAAMVANTDDTFVQFPASLKLQSSNRGERPKEMVDDIEAMLIERLELYQKKNKVLPERVFVFRDGVSEGQFDIVLAEELKGILKAFANPKFKGQGNKAYRPVLSIIICGKRHHARMAPTDAQFADKGGNTRPGTVNDKGITAVYDFDFYLQSHAGIKGHAKGTHYTVVYDENKFTADEIQQGTHNASHLYARATRAVSLVPPAYYADLACERARFYLNNFFDANSEIASTIGSGKGKGKKRSREEEMDLVFQAAVEAWGQGLHPTIRDSMFYQ

>VVO_04305, Argonaute protein, PAZ, IPR003100

MTFNQLNDCTDTSSKGAFKNGISPEPSPSFGHDMLKFFALDQDYINLNHGTFGSVPRPVQDAYTSISDQIESNPDYFGRLLYYRQLDGVRSRLAKLVNAHVDECVIVPNVAYGVNTILRNFEWNEGDIIITTNTAYEAIQHAISYIANRHPHPTTPVFAIQFPTSHANILSSFTAHVKSLVQDSNPTETGIKRKVVAIFDTISSQPGVLWPWKQMVEICREYGVWSVVDAAHSLGQEIDINLKSVDPDFWIANCNKWLYAKRGCALLYVPSRNQAMIKDTLIPALIYPVPGLNPTPFIAKFYWSGSADFAIPLSINAALDFRAYIGGEDKINEYCHQMAIAGGNRLAQILGTSVMDAPNNELTLNMVNVELPLQAKPSGEVWFGLQNKLLEEGRVYAVYYHHNAKWWVRVSSQVYNEVSGIQSKLATIIDPFVLDVRLRKARTSAQGSVPRDHLILVNVGIVLSGSLRVPFIDKQFKWLRRLPERIMSNRGGYRGGGRGGGGGYSGGGGGYSGGGGRGGYGGGGRGRGDFGGGRGGGRGGFGGGAGRGRGGIFAPEQPAIVDKSKTEAETALVLAFKGLKLGDNEIPLRPNFGSAGTDIRLRANYFPINIPKTTFFEYDIQIAPQQGITNKHVKRRIFQLVEATPDWTRLGLKGNVAHDHSAKLVASKQLPQPIDITFPYWDENDNGPTENSKEYTMTIKYIQPIDTEGLINYMQGNQKYRDFDVAPAIAALNLILAAYPSGTGRGIIVGRNKFFFKSPQAIPADLGGGLEAWRGFFSSVRPTFNQLAVNVNVCTTAFYREGELSDVVEAYYRMNGSAKLHSFLKGLRIQLKHLNNRRKTIKAVSKLDANGHKFEYQGAQTSVRQYFKKKYNIELKYPDWELIDVGTPQREDLVPIEVCIVMPGQPYRGKLNDEHTAAMITVAANPPNINANEIVNRGIRELGYDLSGTQNVNLPLNSFKIAIGKHMIPVPARILPTPKIQYYGSSTASIDERASWNLRSLRFTRGAPLSQWVIYVIKDGNTREEFQTMGEVLEVMDKFRDACKAAGLGIGNEQEFKRKQLVKEIRLPPKNVNDPVRKSAGEAIYTAVRGTPFKPPMAIFILSSGDKNVYAAIKRNCDAILDVPSVCMHAAKIRKEKGQPQYMANVALKINMKLGGINHTLAPTSSALQWLQQQPTMLVGIDVTHPGVGSAKGTPSIAAVVASCDQHFAQYPASMELQASKKEMVTNLGKMMVERINHFRKRTTKLPQRIIVYRDGVSEGQFKIVIEEEIPLIYKSFESFNTGETRYRPKLTFVVCGKRHHTRFNPTRAEDADINGNPRPGTVVDRGVTSVYHFDFFLQAHGGLQGTTRPTHYYVLHDDLNFKADELQSLTNSVSYLFGRATKAVSLVSPAYYADLACERGRCYLNTLLSGVSTVQSNVQNYEELVEKEAETLWNRGVSGNLKDTMFYL

>VVO_04091, Argonaute protein, PAZ, IPR003100

MPSPPAKQTKFPRFWKSTSPPRNRPITSGTEDRDSSRLHIRRGTAGSSTSPVSVPAQLEEEPIQILGWRRDFDHEYKPAPDPDINDIFEAEKPFELDEELRDSSLNDLLARFHSMSTSAKDRILRPDFGSGGRPIQLRTNFFRLRVSDKSLYEYNVSITFAKESTDKRLISQRMKQKIFELAEATEAWKGLVDQIAHDYSSTLVSSKELPGESLTITVPFHETNDCRNKEKRAGFRVVMTPSRVLSVGTLLRYLGGDINIQDDEVFPILKALDIILTAPSRRYKQATCVASRRFFFPNDQPKKPEEIGDGLQGRRGFYVGSGIAHRQVVVNIDVSTMAFYSPMQMRDLGFREWEEVRYKLFVKGLRVAVRNTNNSERRGTIHYLYDDKSSDVTLVHPDYFENPRVLSVDKRVTAEYCSVLPNQPFRGILTPQQIKKLIEYATESPAQVARLITADAMHLLSYNASPTLHSFGISVTNDMMSVPGRVIPPPRIRYGRAILDVGPDTRWDPRTVPSTFQSAVKLDKWAVIIISDGDDNPVDQLGENAPLGRKIRTHIDRLLITAFRRMGIDVLKAPLIGVARLPEDHRSDPFRKDVINVIGNVISSMVPAPSFLFFLLANEDRGVYAAIKHKFEVDVGAAHTCMQTKKLLNSDDVKYVLGVAMKVNMKIGGRNQTIICPTLDFLQSKPTLLVGLNVWYPSSHLNTPWIPPVGACASNIDDHFSQFAVSLALQALAQKVMTDLPNMLSTRIMEYKERNGVWPERLIIFRHDIPEEERDKIISAEMKLIYSAFDKITVELPRGKYLPKFALIASSQQHHARFFPANVASAYGHGNPPPGTVVDQGVLSGDKFDFFLQYLSLMIFRP

>VVO_09869, Argonaute protein, PAZ, IPR003100

MPPRAGANPRGQRGGSGPRGARGGGAGRGGPRGGGPGPQHFAAAAPAPSTAGVPAAHVQTVGVKRTGTGTAGRPIQLIVNAFPVSAPESIIYHYDAINPDTLPSRLNLVLIKTLQDDNPLFNPRVVYDGRKNIYSPRELPLGPTSSAQFNVSLSPESSSGRPPKVYTVKLTKVQTINPELLNLFVQGTQSQDNAVLTAIMASNVVTRMAVYDRVASPPVPGTPLFAHNARSTFTNQEVRRIGGGIELWRGFFQSIRPSIGRLIINIDIATGTMYRGGPLLELCLDFLRRPDGDPNILSPKRGLDEPRRRQLQRFLANVRVTAPAPDSGRAQKARPIKGLSNVGADEIRFAQRDGRQTTVARYFAETYRRQLKYPSILCVEIGKGAMFPIEVCIVPPGQIMQKQVPSDLTTSVLEFSTKKPDERLNSIRQGVQALEFGQSEYIRQFGMSINTAGGPIQINARVLQTPALIYNDRSKSRRVTPANGSWNMIDKLLYDPKIITRWVWVIYADERRFRQQQAQDAVRGFIEACQKGGMQVLETDPIIRYENAHGQIANQLRAAGQACASKAQSRGQRGGPDLIVCVLPDNSGDMYTAIKHFGDITMGVATQCLRAFKCGRANMQYWMNVILKVNVKLGGINVIPDPASSFLSEQPTCIMGADVMHPPPGAQGRPSFTALVASVDSNGAKYVPKTCVQTGTVEIIADLKEMVKFGLEKFMQYRQHVEKKGTGLATAPKRLIFYRDGVSEGQFSHVLERELPLIKEACQELGFTPQITLVVVGKRHHFRFIPQNPRDADRSGNCPAGTVVDTGITHPTEWDYYLQSHGGLLGTSRPSHYSVLYDDIGFNPNSIQSLSFALCHVYARATRSVSIPAPVYYADILCARAKNHFAPGSVDMSDTATVLSAEAAQSLETYKRNFRPLHANQEKLMYFSVGGPPCSNITY
