# Supplementary material for: Chilling Stress Triggers VvAgo1-Mediated miRNA-Like RNA Biogenesis in Volvariella volvacea
Source: Front Microbiol. 2020 Sep 15;11:523593. doi: 10.3389/fmicb.2020.523593 (PMC7522536; doi:10.3389/fmicb.2020.523593)
Supplement: TABLE S1 — Primers used for qPCR. [file Data_Sheet_1.zip › Table 7.doc]

**Table S7 Sequence statistics of samples**

| **Sample** | **Raw Reads** | **Clean Reads (≥18 nt)** |
| --- | --- | --- |
| V23-0H1 | 24102842 | 19372288 |
| V23-0H2 | 40898601 | 30426128 |
| V23-0H3 | 53941439 | 42260121 |
| V23-4H1 | 39452709 | 27152026 |
| V23-4H2 | 38668705 | 25860109 |
| V23-4H3 | 36797868 | 20317465 |
| VH3-0H1 | 51379237 | 38689190 |
| VH3-0H2 | 46978881 | 35790072 |
| VH3-0H3 | 24148276 | 20024900 |
| VH3-4H1 | 22858104 | 18450647 |
| VH3-4H2 | 79543571 | 67324584 |
| VH3-4H3 | 32372239 | 26845959 |
