# Supplementary material for: Chilling Stress Triggers VvAgo1-Mediated miRNA-Like RNA Biogenesis in Volvariella volvacea
Source: Front Microbiol. 2020 Sep 15;11:523593. doi: 10.3389/fmicb.2020.523593 (PMC7522536; doi:10.3389/fmicb.2020.523593)
Supplement: TABLE S1 — Primers used for qPCR. [file Data_Sheet_1.zip › Table 9.doc]

**Table S9 Classification statistics of small RNAs (total)**

| **Sample** | **Known miRNA** | **Novel miRNA** | **rRNA** | **snoRNA** | **snRNA** | **tRNA** |
| --- | --- | --- | --- | --- | --- | --- |
| V23-0H1 | 1389 | 300 | 12985174 | 12031 | 65755 | 754917 |
| V23-0H2 | 295 | 258 | 19465933 | 10928 | 75984 | 4100839 |
| V23-0H3 | 529 | 465 | 29112272 | 18036 | 133472 | 3509307 |
| V23-4H1 | 870 | 498 | 14483562 | 19135 | 75409 | 3973952 |
| V23-4H2 | 607 | 732 | 15365083 | 23538 | 77780 | 2916562 |
| V23-4H3 | 1944 | 744 | 13606362 | 30911 | 67034 | 750450 |
| VH3-0H1 | 132 | 175 | 26370880 | 9787 | 63908 | 3841251 |
| VH3-0H2 | 201 | 229 | 28197088 | 13742 | 58160 | 1162265 |
| VH3-0H3 | 127 | 133 | 14558604 | 14449 | 34484 | 763842 |
| VH3-4H1 | 108 | 69 | 13599147 | 15774 | 34391 | 778535 |
| VH3-4H2 | 330 | 200 | 51098667 | 23842 | 123904 | 2829739 |
| VH3-4H3 | 183 | 105 | 21487052 | 12780 | 46035 | 640390 |

*Note:* The first column is the sample name, and the second to the last column is the total number of sequences that are annotated to various types of RNA, respectively.
